# Plasma pentadecanoic acid is modestly related to cardiovascular health in CARDIA and ARIC cohorts: observational associations without evidence of causality

**DOI:** 10.3389/fnut.2026.1720975

**Published:** 2026-02-10

**Authors:** Brian T. Steffen, David R. Jacobs, Aixin Li, Weihong Tang, Daniel Duprez, Mahesh Mathew, Pamela L. Lutsey, So-Yun Yi, Xia Zhou, Lyn M. Steffen

**Affiliations:** 1 Division of Computational Health Sciences, Department of Surgery, School of Medicine, University of Minnesota, Minneapolis, MN, United States; 2 Division of Epidemiology and Community Health, School of Public Health, University of Minnesota, Minneapolis, MN, United States; 3 Cardiovascular Division, Department of Medicine, School of Medicine, University of Minnesota, Minneapolis, MN, United States

**Keywords:** blood pressure, hypertension, Mendelian randomization, odd-chain fatty acid, pentadecanoic acid

## Abstract

**Background:**

Pentadecanoic acid (C15:0) is an odd-chain saturated fatty acid that is being marketed as a heart-healthy supplement.

**Objective:**

To test whether plasma C15:0 is related to cardiovascular outcomes including systolic and diastolic blood pressure (SBP and DBP), functional cardiac measures, and development of hypertension and cardiovascular disease (CVD).

**Methods:**

Plasma phospholipid C15:0 levels were assessed by gas chromatography in 3,196 Coronary Artery Risk Development in Young Adults participants (mean age 45 years; 57% female; 45% Black). Generalized linear models estimated associations of plasma C15:0 with SBP, DBP, and echocardiographic indices. Cox regression estimated risk of incident hypertension (SBP/DBP ≥ 140/90 mmHg or BP medication use) or CVD over a median 10-year period. Covariate adjustments were included to control for likely confounders. Significant associations were tested in a replication subcohort of 3,889 White participants of the Atherosclerosis Risk in Communities Study (ARIC) (mean age 54 years; 52% female) using comparable methods. Two-sample Mendelian randomization (MR) tested for potential causality.

**Results:**

Higher plasma C15:0 levels (per SD) were associated with lower SBP (mm Hg) [*β* = −1.47 (95% CI, −1.99, −0.96)], DBP (mm Hg) [*β* = −1.13 (95% CI, −1.51, −0.74)], and 10-year risk of incident hypertension [hazard ratio = 0.86 (95% CI, 0.78, 0.95)]. These associations were replicated in the ARIC sample. C15:0 levels were not associated with incident CVD in either cohort or with echocardiographic parameters in CARDIA. Two-sample MR analyses provided no evidence for a causal effect of C15:0 on SBP, DBP, resting heart rate, or hypertension.

**Conclusion:**

Observational associations between plasma C15:0 and cardiovascular risk markers were modest but were not supported by cardiac function or MR findings. The collective evidence is not consistent with a causal cardiovascular benefit of C15:0.

## Introduction

Dietary supplement use has increased substantially in the United States over the past two decades, with more than half of adults reporting regular use (1). While supplements like Vitamin-D and creatine have been scrutinized in randomized trials (2–6), others remain poorly characterized, and health claims are based on modest correlations from observational studies or experimental rodent models. Of the latter, pentadecanoic acid (C15:0) is an odd-chain saturated fatty acid primarily derived from dairy fat and has recently been promoted as a “heart-healthy” and “longevity-enhancing” supplement (7).

To date, observational and experimental evidence are consistent with the “heart-healthy” claims. Indeed, observational studies have reported inverse associations between higher circulating C15:0 and risks of heart failure (8) and cardiovascular disease (9–12)—consistent with cardiovascular benefits. Experimental evidence has provided some biological plausibility to these associations whereby C15:0 has been shown to modulate signaling pathways involving mTOR and AMPK (7, 13–15)—proteins implicated in blood pressure (BP) regulation, vascular tone, and hypertension (16–19) as well as cardiac structure and function (20–23). Whether these mechanistic findings translate to humans remains unknown, and no studies have examined associations of C15:0 with outcomes that are downstream of improved BP regulation—cardiac structure or function.

Given the commercialization of C15:0 as a health supplement, its presence in dairy fat, the promising observational and experimental evidence, further investigation is warranted. The present study evaluated whether higher C15:0 is associated with lower systolic and diastolic blood pressure (SBP and DBP), echocardiographic measures of cardiac structure and function, and risks of incident hypertension and cardiovascular disease. To avoid potential bias of estimating intake of a single nutrient based on self-reported dietary data, circulating C15:0 levels were examined as an objective proxy of its intake (12, 24–26). Primary analyses were conducted in the Coronary Artery Risk Development in Young Adults (CARDIA) cohort, with replication in a subcohort of the Atherosclerosis Risk in Communities (ARIC) study. To move beyond correlational inference, we assessed the potential for causality of significant exposure—outcome associations using two-sample Mendelian randomization (MR).

## Methods

### Study populations

The main analysis was conducted in Black and White CARDIA study participants. The CARDIA study cohort was designed to identify factors in young adults that are associated with cardiovascular disease development later in life (27). Participants were recruited between 1985 and 1986 among community samples across four US cities, and 50% of those screened were enrolled. Participants were 18 to 30 years old at baseline and split relatively evenly within each clinic on age, race, sex, and educational attainment at baseline. CARDIA clinic visits were conducted in years 0, 2, 5, 7, 10, 15, 20, 25, 30, and 35. Clinic visits were typically well-attended (>70% of surviving participants) apart from the Y35 exam visit, which was conducted during the COVID-19 pandemic.

A replication analysis was conducted in ARIC participants. The ARIC prospective cohort study was designed to identify risk factors for cardiovascular disease and atherosclerosis (28). Men and women, aged 45–64, were recruited between 1987 and 1989 across four communities in the US (Washington County, Maryland; the northwest suburbs of Minneapolis, Minnesota; Jackson, Mississippi; and Forsyth County, North Carolina). The plasma fatty acid exposure variable was only measured in participants at the Minneapolis field center.

Written informed consent was obtained from all CARDIA and ARIC participants, and all study protocols were approved by the Institutional Review Boards at each participating institution.

### Plasma phospholipid C15:0 measurement

Circulating C15:0 has been widely used as an objective biomarker of dietary intake across numerous cohort studies, particularly as a proxy for dairy fat consumption from milk, yogurt, cheese, and butter (7–12, 24–26). Beef and certain marine foods, including fish skin, are minor contributors (7). C15:0 is incorporated into membrane phospholipids, which exhibit relatively slower turnover compared with other lipid fractions like triacylglycerol but faster turnover than adipocytes (29). When considered with the reproducibility of plasma phospholipid C15:0 over time (intraclass correlation coefficient of ~0.7) (30), plasma phospholipid C15:0 appears to reflect a stable, medium-term dietary exposure over several weeks rather than a proxy of acute intake.

To assess its plasma phospholipid levels, C15:0 was measured at the CARDIA Year 20 exam and at the baseline exam in ARIC in accordance with a standard method described previously (31). Briefly, lipids were obtained from stored (−70 °C) EDTA plasma using a chloroform/methanol extraction method. Triglyceride, cholesterol ester, free fatty acid, and phospholipid fractions were separated by thin layer chromatography. Samples were spiked with heptadecanoic acid (C17:0) prior to measurement and served as an internal control. Phospholipid fatty acids, including C15:0, were harvested and assessed by gas chromatography-flame ionization detection providing a direct biochemical measure of this odd-chain fatty acid. C15:0 levels were examined as a percent of total phospholipid fatty acids in accordance with the detection method.

### BP measurement, incident hypertension and CVD

BP was measured in CARDIA using standardized protocols, detailed in the National Heart Lung and Blood Institute, CARDIA Study Manual of Operation (32). Briefly, trained staff took three readings of SBP and DBP using an Omron oscillometer calibrated to the random zero sphygmomanometer after participants had been seated for 5 min, and readings two and three were averaged to obtain the mean. A 30-s HR was recorded at the radial artery by palpation before the first BP measurement, and pulse pressure was calculated as (SBP − DBP).

Incident CVD was identified in the 10 years following the Year 20 exam in CARDIA and the baseline Visit 1 exam in ARIC. The composite CVD endpoint included coronary heart disease, stroke, or heart failure. Surveillance methods for the CARDIA and ARIC cohorts have been previously described (28, 33). Incident hypertension was identified in the 10 years following the Year 20 exam in CARDIA or the baseline Visit 1 exam in ARIC. Incident hypertension was defined as SBP ≥ 140 mm Hg, DBP ≥ 90 mm Hg (as measured above), or self-reported use of BP medication. These thresholds are higher than those recommended by the 2025 American College of Cardiology/American Heart Association guidelines (SBP ≥ 130 mmHg or DBP ≥ 80 mmHg) (34). We selected the higher cutpoints to (i) increase specificity, i.e., reduce false positives—an important consideration in etiologic biomarker studies (35); (ii) provide a more robust definition to counter the measurement error and reproducibility issues of BP assessment (36, 37); and (iii) ensure comparability with prior cohorts and consistency with historical and international guideline definitions.

### Cardiac function assessment

Analysis of cardiac functional assessment outcomes was only conducted in the CARDIA cohort. The CARDIA cardiac functional assessment was performed by experienced sonographers using Doppler echocardiography and 2-dimensional-guided M-mode echocardiography and an Artida cardiac ultrasound scanner (Toshiba Medical Systems, Japan). Measurements from digitized images were generated using standard off-line image analysis software system (Digisonics, Inc., Houston, Texas). Left ventricular ejection fraction was measured from the apical 4-chamber view per the American Society of Echocardiography guidelines (38). Mitral inflow peak velocities of the early phase (E), late phase (A), and their ratio were estimated using pulsed Doppler echocardiography recordings of transmitral flow. The Advanced Cardiology Package 2D Wall Motion Tracking software system (version 3.0, Toshiba Medical Systems, Tochigi, Japan) estimated circumferential and radial strain measures.

### Covariate data collection

Age, sex, and self-reported race were recorded at the initial visit, Year 0. The highest level of participant education and lifestyle factors including cigarette smoking and alcohol consumption status were assessed with questionnaires at every exam visit. A separate questionnaire was administered to assess physical activity (39), and scores were calculated based on the time spent performing activities and weighted by estimated energy expenditures. For waist circumference, trained personnel took two measurements, and their average served as a metric of adiposity. Finally, a correlational analysis of the plasma fatty acids revealed that myristic acid (C14:0) was moderately associated with C15:0 (*r* = 0.35; *p* < 0.001). Since C14:0 has been positively associated with cardiovascular risk (40, 41), it is included as a covariate here.

### Statistical analysis

Plasma phospholipid fatty acids were measured at the Year 20 exam (*N* = 3,549), which served as the baseline for exposure, outcome, and covariate data. Participants without a plasma fatty acid assessment (*n* = 193), covariate measures (*n* = 155), or reported “unknown” for type 2 diabetes status or hypertension medication use or refused to answer a question were excluded (*n* = 33). This resulted in 3,196 participants for the regression analyses including 886 Black women, 933 White women, 573 Black men, and 804 White men. Replication of significant findings was conducted in an ARIC subcohort using comparable statistical methods. To evaluate the potential for selection bias, we compared demographic and clinical characteristics of the analytic sample versus those who were excluded, and these are presented in Supplementary Table 1. Although some characteristics showed statistical differences between groups, the absolute differences were small and unlikely to meaningfully bias the analytic results. For the ARIC replication, only participants at the Minneapolis, MN field center underwent a plasma fatty acid assessment, and data for 1,875 White men and 2,014 White women were available for analysis.

Regression analyses were conducted using SAS version 9.4 (SAS Institute Inc., Cary, NC). Plasma C15:0 was examined as a continuous variable (per SD). Generalized linear models estimated the associations between plasma C15:0 and continuous outcome variables with adjustments for age, sex, race, field center, education, physical activity, smoking status, alcohol consumption status, waist circumference, fasting glucose, plasma levels of myristic acid (C14:0), self-reported use of BP medications, and prevalent diabetes. BP medication use was controlled for using covariate adjustment rather than imputation-based correction to avoid introducing assumptions about medication-specific treatment effects.

For cardiac functional measurements, a subsample of participants in the main analysis underwent an echocardiography assessment (*N* = 2,336). To control for the number of comparisons for cardiac functional measurements (*n* = 10), we applied a Bonferroni correction, which stipulated a significance threshold of *p* = 0.005. Statistical significance for individual parameter estimates was assessed using two-sided *t*-tests. Since echocardiographic measures were only available in a subset of participants, statistical power was further scrutinized. Based on observed variance in the imaging cohort and the Bonferroni-corrected alpha level to account for the number of outcomes (*α* = 0.005, 80% power), we found that the minimum detectable differences were modest in magnitude—for example, ~2.0 g/m^2^ for LV mass index and ~1.3 percentage points for ejection fraction. It is therefore unlikely that any null results could be attributed to inadequate power.

Cox regression estimated hazard ratios between plasma C15:0 and risks of incident hypertension and CVD with adjustments for age, sex, race, education, field center, plasma C14:0, smoking status, physical activity, alcohol consumption status, waist circumference, prevalent diabetes, and fasting glucose. Those with prevalent hypertension or CVD were excluded from the respective analysis. Statistical significance of associations was assessed using likelihood ratio and Wald chi-square tests. The proportional hazards assumption was evaluated using scaled Schoenfeld residuals, and deviations from proportionality were not detected. Cox models were administratively censored at 10 years to align the risk horizons with the baseline exposure assessment. As an additional consideration for censoring, plasma fatty acids were measured at one timepoint in CARDIA and ARIC participants and are unlikely to be stable over longer periods due to aging and dietary changes.

In CARDIA dietary variables including diet quality and estimated total calories and macronutrient intakes were initially included in regression models, which reduced the analytical sample size by approximately 300 participants. Their inclusion did not materially change the results, so these variables were excluded from the final models.

### Two-sample MR analysis

For the significant associations identified in the regression analyses, MR interrogated potential causality between the plasma C15:0 exposure and identified outcomes. There are three core assumptions of MR analysis that must be acknowledged: (1) relevance, i.e., selected genetic variants must be strong predictors of the exposure of interest, typically evaluated by *F*-statistics >10; (2) independence, i.e., selected genetic variants should not be associated with any confounders of the exposure-outcome relationship; and (3) exclusion restriction, i.e., selected genetic variants only influence the outcome through the exposure of interest.

Genetic instrumental variables associated with plasma C15:0 levels were identified from a genome-wide association study (GWAS) conducted in 8,273 European individuals (42). Clumping was used to prune SNPs in linkage disequilibrium (*r*^2^ < 0.01) within 5 Mb. Because C15:0 is a relatively low-abundance metabolic trait and few variants reached the conventional genome-wide significance threshold (*p* < 5 × 10^−8^), we used a relaxed significance threshold of *p* ≤ 5 × 10^−6^, a strategy used for circulating metabolites to balance instrument strength with sufficient variant number (43, 44). Nine plasma C15:0-associated SNPs were identified under this threshold, and *F*-statistics assessed the presence of weak instrument bias (all SNP *F*-statistics > 20; Supplementary Table 2). The combined instrument explained approximately 2.5% of the variance in plasma C15:0 with a corresponding F-statistic of 23, indicating that substantial weak-instrument bias is unlikely.

Inverse-variance weighted (IVW) random-effects estimates were generated, and sensitivity analyses were conducted including MR-Egger, simple median, and weighted median models. We tested potential causality of the genetic instrument using GWA studies of SBP and DBP (45), resting heart rate (46), and hypertension (47). Given the variance explained by the nine-SNP instrument (~2.5%) and the large outcome GWAS samples, the analyses were well-powered to detect modest associations. For example, the MR analysis examining hypertension (corresponding GWAS sample of ~1.3 million individuals) had >80% power to detect odds ratios on the order of ~1.015 per 1-SD increase in genetically predicted C15:0 (*α* = 0.05). Any undetected relationship was therefore likely to be very small.

## Results

Demographic, lifestyle, and clinical characteristics of 3,196 CARDIA participants are shown across quartiles of plasma C15:0 levels in Table 1. Those with higher plasma C15:0 levels were more likely to be female, White, physically active, non-smokers, have nominally higher education, and showed greater mean levels of plasma C14:0. In addition, those with higher plasma C15:0 showed healthier cardiometabolic indices including lower fasting glucose, waist circumference, and less prevalent diabetes and blood pressure medication use than those with lower C15:0 levels.

**Table 1:** Demographic, lifestyle, and clinical characteristics of 3,196 Coronary Artery Risk Development in Young Adults participants stratified by quartiles of plasma pentadecanoic acid levels.

Characteristic	Quartile of plasma pentadecanoic acid levels (C15:0)				*p*-value
	1 (*N* = 779)	2 (*N* = 811)	3 (*N* = 797)	4 (*N* = 809)	
Plasma C15:0, mean (SD)	0.11 (0.02)	0.16 (0.01)	0.19 (0.01)	0.26 (0.04)	
Age, mean (SD)	45.3 (3.7)	45.1 (3.7)	45.3 (3.6)	45.2 (3.5)	0.51
Male participants, *n* (%)	387 (48.6)	346 (42.2)	363 (44.9)	314 (38.5)	<0.001
Black participants	566 (71.0)	415 (50.7)	256 (31.7)	236 (29.0)	<0.001
Education, median (IQR)	14.0 (12.0, 16.0)	15.0 (13.0, 16.0)	16.0 (13.0, 17.0)	16.0 (14.0, 18.0)	<0.001
Field center					<0.001
Birmingham, AL	288 (36.1)	207 (25.3)	151 (18.7)	121 (14.8)	
Chicago, IL	162 (20.3)	192 (23.4)	175 (21.7)	213 (26.1)	
Minneapolis, MN	154 (19.3)	208 (25.4)	258 (31.9)	218 (26.7)	
Oakland, CA	193 (24.2)	212 (25.9)	224 (27.7)	263 (32.3)	
Smoking status					<0.001
Never	428 (53.7)	506 (61.8)	517 (64.0)	534 (65.5)	
Former	142 (17.8)	162 (19.8)	153 (18.9)	176 (21.6)	
Current	227 (28.5)	151 (18.4)	138 (17.1)	105 (12.9)	
Current drinker	613 (76.9)	639 (78.0)	652 (80.7)	663 (81.3)	0.09
Physical activity, median (IQR)	252 (104, 457)	280 (123, 500)	316 (160, 523)	291 (147, 514)	0.01
Waist circumference (cm), mean (SD)	95.1 (16.6)	93.3 (16.2)	90.8 (14.7)	88.6 (13.9)	<0.001
Fasting glucose, mean (SD)	102.6 (35.9)	97.8 (25.1)	96.1 (19.7)	95.0 (19.9)	<0.001
Prevalent diabetes, *n* (%)	89 (11.2)	66 (8.1)	44 (5.4)	33 (4.0)	<0.001
Prevalent hypertension, *n* (%)	270 (33.9)	177 (21.6)	144 (17.8)	116 (14.2)	<0.001
Taking blood pressure meds, *n* (%)	202 (25.9)	146 (18.0)	113 (14.2)	89 (11.0)	<0.001
Plasma C14:0, mean (SD)	0.21 (0.08)	0.24 (0.07)	0.27 (0.08)	0.29 (0.09)	<0.001

A replication analysis was conducted in an independent cohort of 3,889 White participants from the ARIC study. Characteristics of the ARIC sample are shown in Supplementary Table 3. All ARIC participants were White and were a mean 9 years older than CARDIA participants (54 vs. 45 years of age). Participant characteristics across quartiles of plasma C15:0 in the ARIC subcohort were similar to those of CARDIA. ARIC participants with higher C15:0 levels were more likely to be female, non-smokers, and have greater plasma C14:0 levels as well as lower fasting glucose, waist circumferences, and less prevalent diabetes and blood pressure medication use than those with lower C15:0 levels. In contrast to the pattern found in CARDIA, levels of physical activity were similar across C15:0 quartiles in ARIC.

**Figure 1:**
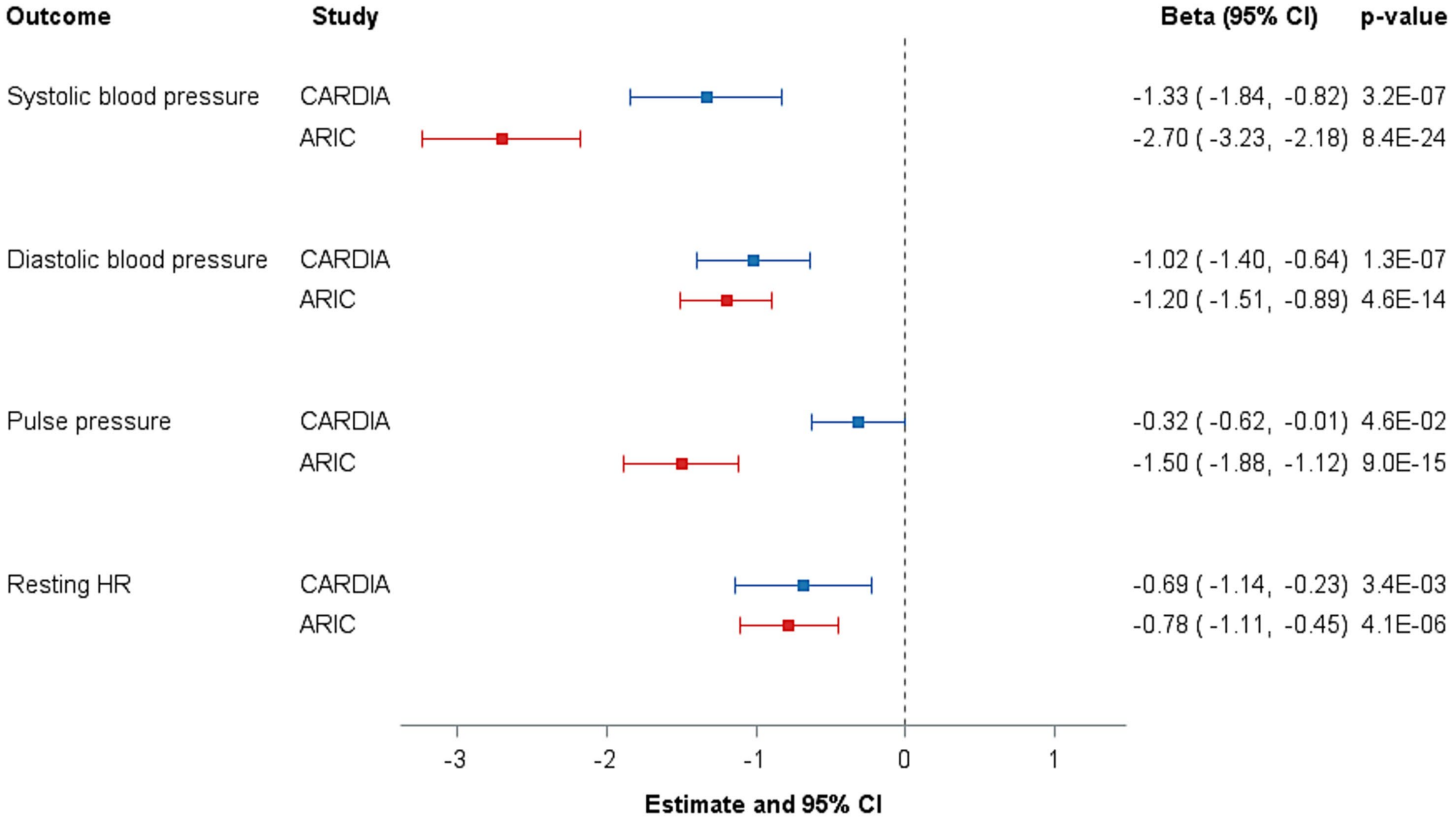
Associations of plasma pentadecanoic acid with blood pressure, pulse pressure, and resting heart rate among CARDIA participants and replication in ARIC cohort participants. Results are presented as beta estimates (95% CI); *p*-values are indicated. Model: Generalized linear regression adjusted for age, sex, race, field center, education, plasma C14:0, current drinking status, smoking status, waist circumference, physical activity, prevalent diabetes, and BP medication use. CARDIA, Coronary Artery Risk Development in Young Adults; ARIC, Atherosclerosis Risk in Communities Study; SD, standard deviation; ref., referent; SBP, systolic blood pressure; DBP, diastolic blood pressure.

Associations between plasma C15:0 and SBP, DBP, pulse pressure, and resting heart rate are shown in Figure 1. In the CARDIA sample, plasma C15:0 (per SD) was related to lower SBP (−1.47 mm Hg; *p* = 2.6×10^−8^), DBP (−1.13 mm Hg; *p* = 8.3×10^−9^), pulse pressure (−0.35 mm Hg; *p* = 0.03), and resting heart rate (−0.71 beats per minute; *p* = 0.03). Greater magnitudes of associations were found in the ARIC sample for SBP (per SD) (−2.69 mm Hg; *p* = 1.8×10^−23^), DBP (−1.18 mm Hg; *p* = 1.2×10^−13^), and pulse pressure (−1.50 mm Hg; *p* = 9.0×10^−15^), but the association for resting heart rate was similar to the CARDIA sample (−0.77 beats per minute; *p* = 5.3×10^−6^). Given the larger proportion of Black individuals with lower C15:0 levels, race interactions were tested in the CARDIA sample and found to be non-significant (all *p* > 0.05).

**Figure 2:**
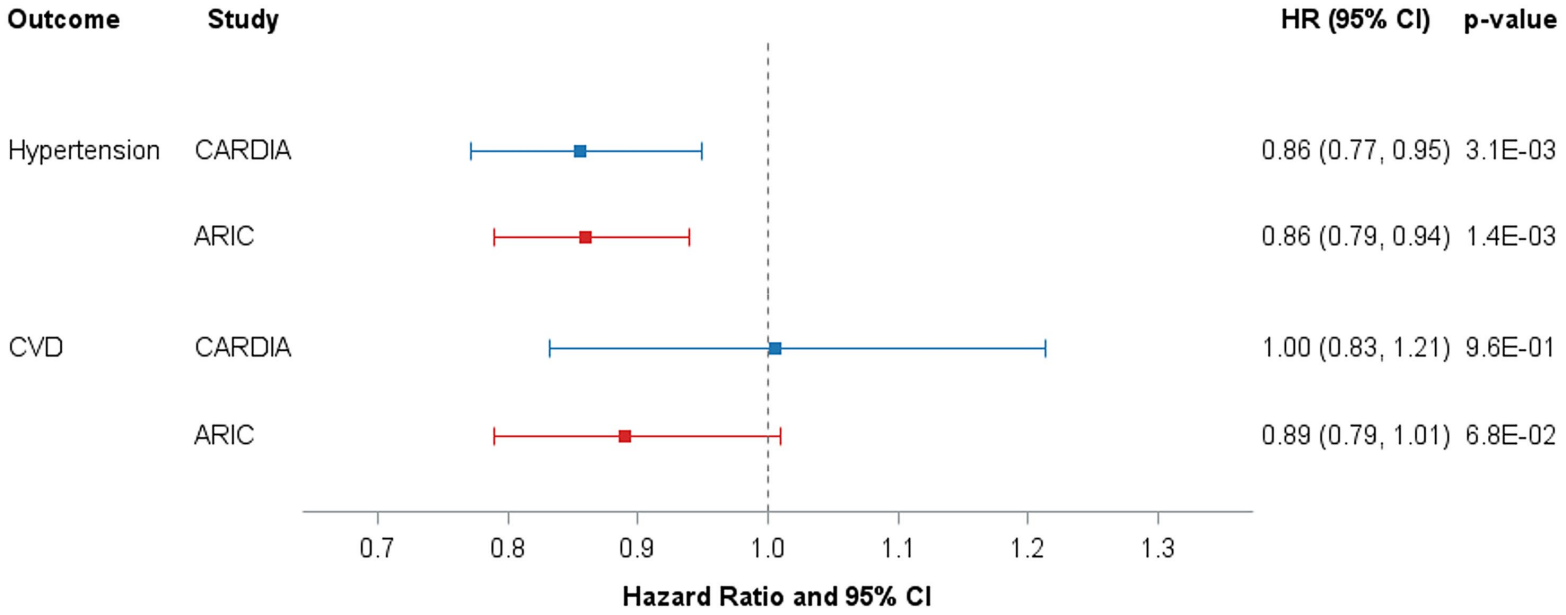
Risks of incident hypertension and CVD per SD of plasma pentadecanoic acid among CARDIA and ARIC participants over a median 10-year follow-up. Results are presented as hazard ratios (95% CI), and *p*-values are indicated. Model: Cox proportional hazards regression adjusted for age, sex, race, field center, education, plasma C14:0, current drinking status, smoking status, waist circumference, physical activity, prevalent diabetes, and fasting glucose. CARDIA, Coronary Artery Risk Development in Young Adults; ARIC, Atherosclerosis Risk in Communities Study; SD, standard deviation.

Prospective associations of plasma C15:0 and risks of incident hypertension and CVD are shown in Figure 2. For incident hypertension, there were 535 cases in CARDIA and 684 in ARIC. Over median 10-year follow-up periods, greater C15:0 (per SD) levels were associated with approximately 14% lower risks of incident hypertension in CARDIA and ARIC samples. For incident CVD, there were 140 cases in CARDIA and 316 cases in ARIC over their respective median 10-year follow-up windows. Greater C15:0 (per SD) was not significantly associated with incident CVD in either cohort. Race interactions for prospective associations were non-significant in the CARDIA sample (all *p* > 0.05).

For the measures of cardiac function, no associations were evident after adjustment for multiple comparisons (all *p* > 0.005), and 8/10 associations did not reach a significance level of *p ≤* 0.05. Results are shown in Supplementary Table 4.

To test for potential causality between C15:0 and outcomes that reached significance, two-sample MR analyses were performed. Relationships between the C15:0 genetic instrument and SBP, DBP, resting heart rate, and hypertension were tested, and no evidence of causality was observed (Table 2). Directional pleiotropy was not evident based on MR-Egger intercepts (all *p* > 0.05). Methods comparisons across exposure variables show that sensitivity analyses MR-Egger, weighted median, and MR-PRESSO analyses were consistent with the IVW models in terms of non-significant findings (all *p*-values > 0.05).

**Table 2:** Results for the two-sample Mendelian randomization analysis of plasma pentadecanoic acid with cardiovascular outcomes^a^.

Outcome	Method	Estimate	95% CI	*p*-value	N SNP
SBP	IVW	−0.306	−1.087 to 0.476	0.44	9
	Weighted median	−0.050	−0.424 to 0.324	0.79	9
	MR-Egger (intercept)	0.700	−1.223 to 2.623	0.48	9
		−0.118	−0.325 to 0.089	0.26	9
	MR-PRESSO (raw)	−0.306	−1.248 to 0.637	0.47	9
	MR-PRESSO (outlier-corrected)	−0.064	−0.455 to 0.327	0.69	8
DBP	IVW	−0.175	−0.779 to 0.43	0.57	9
	Weighted median	0.087	−0.143 to 0.316	0.46	9
	MR-Egger (intercept)	0.946	−0.391 to 2.283	0.17	9
		−0.132	−0.276 to 0.012	0.07	9
	MR-PRESSO (raw)	−0.175	−0.904 to 0.555	0.59	9
	MR-PRESSO (outlier-corrected)	−0.019	−0.25 to 0.213	0.83	7
Resting HR	IVW	0.159	−0.082 to 0.40	0.19	8
	Weighted median	0.196	−0.093 to 0.485	0.18	8
	MR-Egger (intercept)	−0.327	−0.883 to 0.228	0.25	8
		0.062	−0.003 to 0.127	0.06	8
	MR-PRESSO (raw)	0.159	−0.141 to 0.46	0.24	8
	MR-PRESSO (outlier-corrected)	NA^b^	NA	NA	8
Hypertension	IVW	0.009	−0.041 to 0.059	0.73	9
	Weighted median	0.023	−0.015 to 0.060	0.24	9
	MR-Egger (intercept)	0.064	−0.065 to 0.193	0.33	9
		−0.007	−0.021 to 0.008	0.37	9
	MR-PRESSO (raw)	0.009	−0.051 to 0.069	0.73	9
	MR-PRESSO (outlier-corrected)	0.03	−0.009 to 0.068	0.10	8

Estimates (95% confidence intervals) for inverse variance weighted, MR-Egger, weighted median, and MR-PRESSO.

a Outcomes that reached statistical significance in observational analyses were tested using MR.

b No outlier detected.

## Discussion

In this community-based cohort study of approximately 3,200 Black and White men and women, plasma levels of C15:0 were independently associated with modestly lower SBP, DBP, pulse pressure, and resting heart rate, and a 10% lower 10-year risk of incident hypertension after adjustment for multiple covariates. These results were replicated in an independent cohort of 3,889 White male and female ARIC participants. By contrast, plasma C15:0 was not related to echocardiography-derived measures of cardiac anatomy, systolic and diastolic functions, or incident CVD. Results of MR analyses indicated that genetically higher plasma C15:0 levels were unrelated to any of the replicated associations and provided no evidence of a causal relationship.

### C15:0 and health outcomes: interventional, observational, and MR evidence

C15:0 is currently marketed for its cardiovascular, liver, metabolic health benefits as well as longevity. Partially confirming the health claims, a recent randomized, double-blinded, and placebo-controlled trial tested 200 mg of C15:0 on multiple outcomes (48). Despite this low dose (by comparison, fish oil fatty acids are often prescribed for hypertriglyceridemia at 4,000 mg/day), C15:0 was shown to reduce plasma alanine and aspartate transferases over 12 weeks, but only in “responders” whose post-treatment plasma levels reached >5 μg/mL.

Based on this trial and the claims of cardiovascular benefits of C15:0, we aimed to conduct a rigorous observational study to examine cardiovascular outcomes and follow-up any positive results with an MR analysis. Indeed, our initial observational findings were robust across two independent cohorts. The associations with SBP, DBP, and incident hypertension were significant, directionally consistent, and fulfilled several Bradford Hill criteria for inferring causality including consistency (between CARDIA and ARIC cohorts), temporality (association with incident hypertension), biological plausibility (through its putative effects on AMPK and eNOS activation) (17, 18), and coherence (agreement with previous associations of heart failure (8) and CVD (9–11)). Moreover, our findings were in agreement with a recent cross-sectional study that reported inverse associations between C15:0 and hypertension prevalence (49). And yet, positive associations were not supported by the cardiac imaging or MR analyses. Indeed, no cardiac structure or function outcomes were related to C15:0, which is inconsistent with any clinically meaningful association of C15:0 on blood pressure or hypertension risk. The results of the MR analyses were also null and inconsistent with any cardiovascular benefit of plasma C15:0.

The discrepancy between the positive observational associations and the null genetic findings likely reflects residual confounding in the observational models. Specifically, participants with higher C15:0 levels had healthier cardiometabolic profiles—lower waist circumference, lower diabetes prevalence, lower proportions of smokers, and lower fasting glucose (Table 1). Despite statistical adjustment, residual and unmeasured confounding of this type is difficult to eliminate in nutritional epidemiology. The overall evidence is therefore more consistent with C15:0 serving as a biomarker of healthier lifestyle and metabolic status rather than a biologically active determinant of blood pressure or hypertension risk.

It must be acknowledged that the observational associations reflect a genuine biological relationship, and sources of bias skewed the MR results. Supporting this possibility, we used a relaxed significance threshold (*p* ≤ 5×10^−6^) to generate a nine-SNP genetic instrument for the exposure variable. However, each SNP demonstrated adequate strength (all *F* > 20), and the instrument explained 2.5% of variance in plasma C15:0, which is relatively robust for a metabolite-level exposure (42, 50). These make the possibility of a weak instrument less likely. Apart from a weak instrument bias, horizontal pleiotropy may have been present whereby the genetic instrument influences the outcomes via pathways *other than through C15:0*. This can theoretically attenuate estimates and conceal a causal effect(s). However, we found no evidence for pleiotropy in the y-intercepts of the MR-Egger sensitivity analyses or in the outlier-corrected MR-PRESSO results. Finally, bias in the MR analyses would not account for the absence of associations between plasma C15:0 and the echocardiography outcomes. This triangulation of evidence—observational analyses of blood pressure outcomes, cardiac imaging, and MR results—failed to reveal a coherent pattern that supports a biological effect of plasma C15:0 on cardiovascular health. Considered together, the evidence is more consistent with C15:0 as a correlational biomarker of healthier lifestyle patterns and challenges claims by commercial entities promoting C15:0 as a cardioprotective supplement.

### Strengths and limitations

In terms of strengths, the main analysis included over 3,000 Black and White men and women from four field centers across the US, and the replication featured over 3,800 White men and women at one US field center. The C15:0 exposure variable was objectively measured instead of estimating its intake from diet questionnaires. An examination of correlations among plasma phospholipid fatty acids revealed that myristic acid (C14:0) was moderately associated with C15:0 (*r* = 0.35), and it was included as a covariate to control for likely confounding. Other dietary variables such as diet quality and total caloric intake were considered as potential confounders; however, these measures were not available in all participants and including them would have reduced the analytic sample size. Given their minimal impact on effect estimates in sensitivity analyses, we excluded them from primary models to preserve statistical power. Finally, two-sample MR analyses were used to test for potential causality between the C15:0 exposure and cardiovascular outcomes for significant exposure—outcomes associations.

The present study also has several limitations. First, C15:0 represents a fraction of total phospholipid fatty acids (<0.5%), which increases susceptibility to measurement error and imprecision compared to more abundant fatty acids. Second, heptadecanoic acid (C17:0)—another odd-chain saturated fatty acid often analyzed alongside C15:0—served as an internal standard in the gas chromatography assay. Consequently, its concentration could not be quantified as an independent biomarker for this analysis. Third, the MR analyses were conducted using GWA studies in those of European ancestry. While this limits sources of bias including the potential for linkage disequilibrium artifacts and population stratification, it does not eliminate them and also limits generalizability. In addition, and as discussed above, the nine-SNP instrument explained ~2.5% of the variance in plasma C15:0, so the MR analyses were powered to detect moderate to strong causal effects, but we cannot exclude the presence of small associations. Finally, the generalizability of the replication findings is limited by the availability of fatty acid measurements in the Minneapolis field center in ARIC, which provided a replication sample exclusively in White participants. Although the demographic and cardiometabolic characteristics of this subset are broadly comparable to the larger ARIC cohort and race interactions in CARDIA were nonsignificant, the absence of Black participants in the replication sample should be acknowledged.

## Conclusion

Higher plasma C15:0 levels were modestly associated with lower SBP and DBP as well as 10-year risk of incident hypertension, but these findings were not supported by associations with cardiac function, incident CVD, or MR analyses. Considered together, these results are inconsistent with a cardiovascular benefit and instead suggest that C15:0 may act as a biomarker of cardiometabolic health.

## Data Availability

The datasets presented in this article are not readily available due to participant confidentiality. CARDIA and ARIC datasets can be obtained by submitting a proposal to respective P&P committees. Requests to access these datasets should be directed to ARIC: aricjhu@jhu.edu; aricpub@unc.edu; CARDIA: https://sites.uab.edu/cardia/.
