# Supplementary material for: Plasma pentadecanoic acid is modestly related to cardiovascular health in CARDIA and ARIC cohorts: observational associations without evidence of causality
Source: Front Nutr. 2026 Feb 10;13:1720975. doi: 10.3389/fnut.2026.1720975 (PMC12931280; doi:10.3389/fnut.2026.1720975)
Supplement: Supplementary file 1 [file Data_Sheet_1.DOCX]

**Supplemental Material**

Supplemental Table 1. Demographic and clinical characteristics of the analytic sample versus participants excluded due to missingness.

| **Factor** | **Analytic sample** | **Missing data** | **p-value** |
| --- | --- | --- | --- |
|  | **(N=3196)** | **(N=354)** |  |
| C15:0, mean (SD) | 0.18 (0.06) | 0.17 (0.06) | 0.004 |
| C14:0, mean (SD) | 0.25 (0.08) | 0.27 (0.09) | 0.004 |
| Age, mean (SD) | 45.21 (3.61) | 45.16 (3.82) | 0.8 |
| Male, n (%) | 1377 (43.1) | 159 (44.9) | 0.51 |
| Black participants, n (%) | 1459 (45.7) | 193 (54.5) | 0.002 |
| Smoking status, n (%) |  |  |  |
| Never | 1961 (61.4) | 189 (59.1) | 0.15 |
| Former | 626 (19.6) | 56 (17.5) |  |
| Current | 609 (19.1) | 75 (23.4) |  |
| Current drinker, n (%) | 2533 (79.3) | 203 (72.8) | <0.001 |
| PA, median (Q1, Q3) | 276 (126, 487.5) | 228 (103, 409) | 0.001 |
| WC, mean (SD) | 91.77 (15.51) | 93.56 (15.95) | 0.04 |
| Fasting glucose, mean (SD) | 97.35 (24.43) | 105.12 (41.54) | <0.001 |
| Systolic BP, mean (SD) | 115.5 (14.6) | 118.2 (15.2) | <0.001 |
| Diastolic BP, mean (SD) | 72.1 (11.1) | 73.9 (11.6) | 0.004 |
| Prevalent HTN, n (%) | 745 (23.3) | 94 (26.6) | 0.09 |
| Incident HTN (10-year), n (%) | 1029 (38.4) | 114 (42.2) | 0.22 |

Abbreviations: PA=physical activity; WC=waist circumference; BP=blood pressure; HTN=hypertension

Supplemental Table 2. SNP components characteristics of the pentadecanoic acid genetic instrument including the variant chromosomal positions, alleles, and F-statistics are shown below.

| **SNP** | **Chr** | **Position** | **Effect allele** | **Other allele** | **Effect allele frequency** | ***p-*value** | **F-statistic** | **Exposure N** |
| --- | --- | --- | --- | --- | --- | --- | --- | --- |
| rs10933724 | 3 | 194249062 | C | G | 0.142 | 4.4E-06 | 21.1 | 8273 |
| rs113915691 | 19 | 56248382 | A | G | 0.063 | 1.7E-07 | 27.4 | 8273 |
| rs116530753 | 4 | 156785921 | A | G | 0.050 | 5.4E-07 | 25.1 | 8273 |
| rs13098128 | 3 | 47756066 | A | C | 0.680 | 3.8E-06 | 21.4 | 8273 |
| rs1971863 | 1 | 159669141 | C | T | 0.254 | 1.4E-06 | 23.3 | 8273 |
| rs2819083 | 6 | 156379682 | A | G | 0.378 | 3.3E-06 | 21.6 | 8273 |
| rs7543700 | 1 | 63122765 | G | T | 0.035 | 2.9E-06 | 21.9 | 8273 |
| rs79507970 | 12 | 24993537 | C | G | 0.020 | 2.6E-06 | 22.1 | 8273 |
| rs79859092 | 5 | 96814807 | C | T | 0.036 | 4.3E-06 | 21.1 | 8273 |

Supplemental Table 3. Demographic, lifestyle, and clinical characteristics of Atherosclerosis Risk in Communities Study participants stratified by quartiles of plasma pentadecanoic acid levels.

| **Characteristic** | **Quartile of plasma pentadecanoic acid levels (C15:0)** | | | | ***p*-value** |
| --- | --- | --- | --- | --- | --- |
|  | 1 | 2 | 3 | 4 |  |
|  | N=1078 | N=758 | N=1100 | N=995 |  |
| Plasma C15:0 (%), mean (SD) | 0.12 (0.02) | 0.16 (0.01) | 0.18 (0.01) | 0.22 (0.02) |  |
| Age, mean (SD) | 54.5 (5.5) | 54.4 (5.7) | 53.6 (5.6) | 53.5 (5.7) | <0.001 |
| Male, n (%) | 621 (57.6) | 372 (49.1) | 512 (46.5) | 386 (38.8) | <0.001 |
| Education, n (%) |  |  |  |  | <0.001 |
| Less than HS | 81 (7.5) | 49 (6.5) | 58 (5.3) | 62 (6.2) |  |
| HS diploma or GED | 430 (39.9) | 256 (33.8) | 366 (33.3) | 307 (30.9) |  |
| Vocational school | 148 (13.7) | 87 (11.5) | 132 (12.0) | 120 (12.1) |  |
| Some college | 203 (18.8) | 167 (22.1) | 247 (22.5) | 218 (21.9) |  |
| Undergraduate degree | 135 (12.5) | 123 (16.2) | 182 (16.5) | 162 (16.3) |  |
| Professional/graduate school | 80 (7.4) | 75 (9.9) | 115 (10.5) | 125 (12.6) |  |
| Smoking status, n (%) |  |  |  |  | <0.001 |
| Current | 308 (28.8) | 187 (24.9) | 201 (18.3) | 183 (18.5) |  |
| Former | 491 (45.8) | 317 (42.3) | 449 (41.0) | 330 (33.4) |  |
| Never | 272 (25.4) | 246 (32.8) | 446 (40.7) | 474 (48.0) |  |
| Current drinker, n (%) |  |  |  |  | <0.001 |
| Current | 973 (90.8) | 634 (84.5) | 871 (79.5) | 763 (77.3) |  |
| Former | 75 (7.0) | 91 (12.1) | 173 (15.8) | 158 (16.0) |  |
| Never | 23 (2.1) | 25 (3.3) | 52 (4.7) | 66 (6.7) |  |
| Waist circumference, mean (SD) | 96.8 (13.6) | 95.3 (13.2) | 94.4 (13.0) | 92.8 (13.1) | <0.001 |
| Total PA (METS/wk), median (Q1, Q3) | 542 (0, 1104) | 577 (0, 1166.9) | 608 (53, 1213) | 605 (44, 1138) | 0.30 |
| Plasma C14:0, mean (SD) | 0.23 (0.07) | 0.24 (0.07) | 0.26 (0.07) | 0.31 (0.08) | <0.001 |
| Fasting glucose, mean (SD) | 6.1 (1.9) | 6.0 (1.9) | 5.7 (1.4) | 5.6 (1.2) | <0.001 |
| Prevalent hypertension, n (%) | 358 (33.4) | 187 (24.7) | 248 (22.6) | 199 (20.1) | <0.001 |
| Prevalent diabetes, n (%) | 81 (7.5) | 48 (6.3) | 45 (4.1) | 34 (3.4) | <0.001 |
| BP medication use, n (%) | 307 (28.5) | 184 (24.3) | 242 (22.0) | 185 (18.6) | <0.001 |

Definitions: ARIC=Atherosclerosis Risk in Communities; PA=physical activity; C14:0=myristic acid; BP=blood pressure

Supplemental Table 4. Associations of plasma pentadecanoic acid (per SD) with echocardiography-derived cardiac function measures (log-transformed) among CARDIA participants presented as beta estimates (95% CI); *p*-values are indicated.

| **Echocardiography Outcome** | **Estimate (95% CI)** | ***p-*value** | **N** |
| --- | --- | --- | --- |
| Mitral valve peak E-wave to A-wave ratio | 0.014 (0.002, 0.025) | 0.02 | 2311 |
| Left ventricular index | -0.003 (-0.013, 0.008) | 0.62 | 2310 |
| Left ventricular ejection fraction | 0.003 (-0.003, 0.008) | 0.29 | 2311 |
| Lateral E-wave prime velocity | 0.004 (-0.007, 0.014) | 0.51 | 2293 |
| Mitral valve deceleration time | -0.004 (-0.016, 0.007) | 0.44 | 2306 |
| Mitral valve peak A-wave velocity | -0.012 (-0.023, -0.001) | 0.03 | 2311 |
| Mitral valve peak E-wave velocity | 0.002 (-0.007, 0.012) | 0.62 | 2311 |
| e | 0.002 (-0.007, 0.012) | 0.62 | 2289 |
| HEEP | 0.00 (-0.012, 0.011) | 0.96 | 2301 |
| 4CH longitudinal peak strain (%)* | -0.018 (-0.126, 0.091) | 0.75 | 2095 |

Definitions: CARDIA=Coronary Artery Risk Development in Young Adults; SD=standard deviation; LVEF=left ventricular ejection fraction; HEEP=mitral valve peak E-wave velocity /mean of lateral E-wave prime velocity and septal E-wave prime velocity

*4CH longitudinal peak strain was not log-transformed
